# Supplementary material for: A curated C. difficile strain 630 metabolic network: prediction of essential targets and inhibitors
Source: BMC Syst Biol. 2014 Oct 15;8:117. doi: 10.1186/s12918-014-0117-z (PMC4207893; doi:10.1186/s12918-014-0117-z)
Supplement: Additional file 1: Figure S1. — Comparison of the metabolites (A) and genes (B) from iMLTC806cdf to those in the automatic network. Figure S2. Comparison of the metabolites (A), genes (B) and reaction (C) from the Clostridium difficile metabolic network to those from the network of Clostridium acetobutylicum. Figure S3. Example of reaction involving oxygen and their curation. Figure S4. Stickland reactions. Stickland reactions are an example of reactions often incomplete in metabolic databases that were added to the network based on Clostridium difficile specific literature. Table S1. Validation: addition or removal of various metabolites and comparison with literature data. Table S2. Utilization of various metabolites as carbon source and comparison with literature data. Table S3. List of dead-end metabolites identified in Clostridium acetobutilicum network (iCAC490). Table S4. Predicted Clostridium difficile gene essentiality vs. experimental data for functional homologs in Bacillus subtilis. Table S5. Comparison of predicted C. difficile and C. acetobutylicum gene essentiality. Table S6. Details of the predicted essential genes and comparison with targets identified in other target databases. Table S7. Binding site environment similarity used to find possible cross-reactive targets in human for Clostridium difficile essential genes without human homologs. Table S8. List of essential gene pairs with (A) or without (B) synergetic effect. Table S9. Details of the double mutant deleterious gene pairs with synergetic effect. Table S10. Functional classification of metabolic pathways. Table S11. Distribution of essential genes and genes involved in essential pairs in pathways. Table S12. Treatment of dead-end metabolites in the network. Table S13. Full list of protein complexes present in the network. Table S14. Clostridium difficile macromolecule and biomass constitution. [file 12918_2014_117_MOESM1_ESM.zip › Additional files/Table_S4.docx]

**Table S4. Predicted *Clostridium difficile* gene essentiality vs. experimental data for functional homologs in *Bacillus subtilis^a^***.

| *C. difficile* | Lethal | Non-lethal | Total |
| --- | --- | --- | --- |
| *B. subtilis* |  |  |  |
| Lethal | 45 (42) | 45 (35) | 90 (77) |
| Non-lethal | 22 (23) | 506 (425) | 528 (448) |
| Total | 67 (65) | 551 (460) | 618 (525) |

^a^ Values in parenthesis represent sequence homologs.
